# Identification of Antibacterial Sterols from Korean Wild Mushroom *Daedaleopsis confragosa* via Bioactivity- and LC-MS/MS Profile-Guided Fractionation

**DOI:** 10.3390/molecules27061865

**Published:** 2022-03-14

**Authors:** Myung Woo Na, Eunjin Lee, Dong-Min Kang, Se Yun Jeong, Rhim Ryoo, Chul-Young Kim, Mi-Jeong Ahn, Kyo Bin Kang, Ki Hyun Kim

**Affiliations:** 1 School of Pharmacy, Sungkyunkwan University, Suwon 16419, Korea; myeong500@naver.com (M.W.N.); kdm7105@gnu.ac.kr (S.Y.J.); 2 Research Institute of Pharmaceutical Sciences, College of Pharmacy, Sookmyung Women’s University, Seoul 04310, Korea; dmswls8180@sookmyung.ac.kr; 3 College of Pharmacy and Research Institute of Pharmaceutical Sciences, Gyeongsang National University, Jinju 52828, Korea; dlawktkark@naver.com (D.-M.K.); chulykim@hanyang.ac.kr (M.-J.A.); 4 Special Forest Products Division, Forest Bioresources Department, National Institute of Forest Science, Suwon 16631, Korea; rryoo@korea.kr; 5 College of Pharmacy, Hanyang University, Ansan 15588, Korea; amj5812@gnu.ac.kr

**Keywords:** *Daedaleopsis confragosa*, Polyporaceae, ergostane-type steroids, LC-MS/MS, anti-*H. pylori* activity

## Abstract

As part of an ongoing natural product chemical research for the discovery of bioactive secondary metabolites with novel structures, wild fruiting bodies of *Daedaleopsis confragosa* were collected and subjected to chemical and biological analyses. We subjected the fractions derived from the methanol extract of the fruiting bodies of *D. confragosa* to bioactivity-guided fractionation because the methanol extract of *D. confragosa* showed antibacterial activity against *Helicobacter pylori* strain 51, according to our bioactivity screening. The *n*-hexane and dichloromethane fractions showed moderate to weak antibacterial activity against *H. pylori* strain 51, and the active fractions were analyzed for the isolation of antibacterial compounds. Liquid chromatography-tandem mass spectrometry (LC–MS/MS) analysis revealed that the *n*-hexane fraction contains several compounds which are absent in the other fractions, so the fraction was prioritized for further fractionation. Through chemical analysis of the active *n*-hexane and dichloromethane fractions, we isolated five ergosterol derivatives (**1**–**5**), and their chemical structures were determined to be demethylincisterol A_3_ (**1**), (20*S*,22*E*,24*R*)-ergosta-7,22-dien-3β,5α,6β-triol (**2**), (24*S*)-ergosta-7-ene-3β,5α,6β-triol (**3**), 5α,6α-epoxy-(22*E*,24*R*)-ergosta-7,22-dien-3β-ol (**4**), and 5α,6α-epoxy-(24*R*)-ergosta-7-en-3β-ol (**5**) by NMR spectroscopic analysis. This is the first report on the presence of ergosterol derivatives (**1**–**5**) in *D. confragosa*. Compound **1** showed the most potent anti-*H. pylori* activity with 33.9% inhibition, rendering it more potent than quercetin, a positive control. Compound **3** showed inhibitory activity comparable to that of quercetin. Distribution analysis of compound **1** revealed a wide presence of compound **1** in the kingdom Fungi. These findings indicate that demethylincisterol A_3_ (**1**) is a natural antibiotic that may be used in the development of novel antibiotics against *H. pylori*.

## 1. Introduction

Mushrooms have been used for treating various diseases, including diabetes, cancer, and arteriosclerosis, in oriental medicine [1]. In addition, in recent decades, numerous pharmacological and phytochemical studies on mushrooms have proven that they are rich sources of novel compounds with various desirable pharmacological activities such as anti-mutagenic, antioxidant, immunomodulatory, and angiostatic activities, as well as cytotoxicity against cancer cells [1,2,3,4,5,6,7]. Based on this experimental evidence, mushrooms have emerged as potential natural sources for the discovery of bioactive lead molecules; however, most studies have focused on well-known edible ones and little is known regarding biologically active secondary metabolites derived from wild or inedible mushrooms.

As part of our continuing natural product discovery program for the identification of novel bioactive compounds from miscellaneous natural resources [8,9,10,11,12,13,14], we continued chemical and biological analyses of methanolic extracts of the fruiting bodies of *D. confragosa*, because the methanol extract of *D. confragosa* showed antibacterial activity against *Helicobacter pylori* strain 51 in our bioactivity screening. *D. confragosa* has rarely been investigated in terms of its chemical constituents. In our recent untargeted metabolomics study on 40 species of Polyporaceae, *Daedaleopsis tricolor* was highlighted as one of the promising producers of unique specialized metabolites [15]. As the appearance of *D. confragosa* is similar to that of *D. tricolor* and a recent ITS sequences-based phylogenetic analysis suggested that *D. confragosa* and *D. tricolor* may be two morpho-ecological varieties of one species [16], consistent with the suggestion of a mitochondrial small subunit rDNA sequences-based study in 1999 [17], we hypothesized that *D. confragosa* would show unique specialized metabolites as *D. tricolor*.

We applied the fractions derived from the methanol extract of *D. confragosa* into bioactivity-guided fractionation and observed that the *n*-hexane and CH_2_Cl_2_ fractions showed moderate to weak antibacterial activity against *H. pylori* strain 51. Based on the screening results, we aimed to isolate antibacterial compounds from the active *n*-hexane and CH_2_Cl_2_ fractions, aided by a liquid chromatography-tandem mass spectrometry (LC–MS/MS)-based chemical profiling. Herein, we describe the chemical profile- and bioactivity-guided fractionation of the fractions, isolation and structural determination of compounds **1**–**5**, and evaluation of their anti-*H. pylori* activity.

## 2. Results and Discussion

### 2.1. Extraction of Metabolites from D. confragosa and Bioactivity-Guided Fractionation

Dried *D. confragosa* fruiting bodies were extracted with 80% methanol under reflux, yielding the crude methanol extract by rotary evaporation. The resultant methanol extract was sequentially subjected to solvent partitioning with four organic solvents, namely *n*-hexane, dichloromethane (CH_2_Cl_2_), ethyl acetate (EtOAc), and *n*-butanol (BuOH). Thus, four main solvent fractions with increasing polarity were obtained: *n*-hexane, CH_2_Cl_2_, EtOAc, and BuOH-soluble fractions. The anti-*H. pylori* activity of the fractions was evaluated using a clinical strain of *H. pylori* 51. Although the four fractions showed weak or mild inhibitory activity against *H. pylori* strain 51, *n*-hexane- and CH_2_Cl_2_-soluble fractions showed more potent and similar inhibitory activity, respectively, than the positive control, quercetin (Table 1).

### 2.2. LC–MS/MS Analysis of the Extract and Fractions

**Figure 1:**
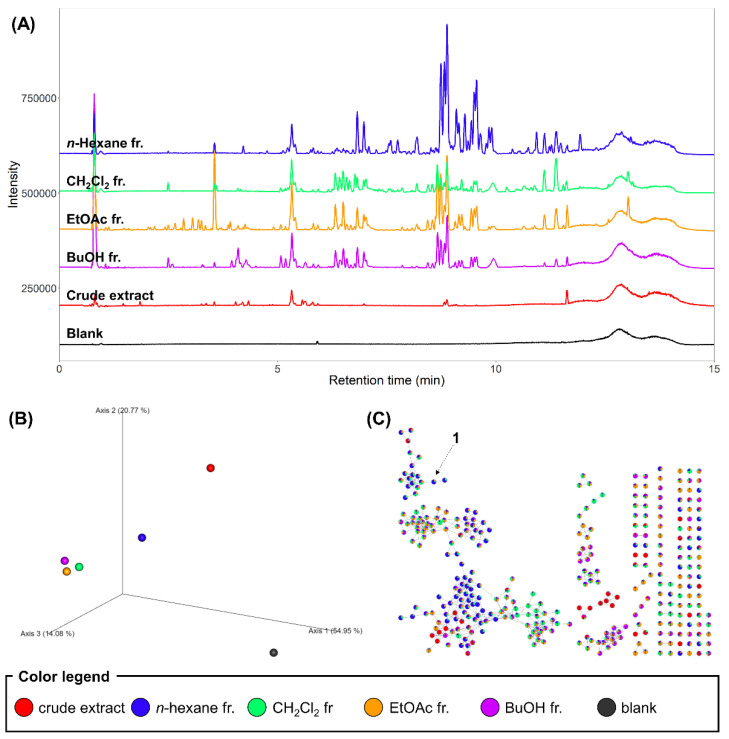
(**A**) LC–MS base peak ion (BPI) chromatograms of the crude extract and fractions of *D. confragosa*. Gaps between chromatograms were added for visualization of their difference; therefore, the y-axis values are not equal to the absolute intensities. (**B**) PCoA plot based on the Bray–Curtis similarity between the MS data of the crude extract and fractions of *D. confragosa*. (**C**) MS/MS spectral network of the crude extract and fractions of *D. confragosa*. The spectral node representing compound **1** is highlighted.

As the crude methanol extract and fractions showed different bioactivities, we performed chemical profiling of the samples to obtain insights into the bioactive components. Contrary to our hypothesis, MS ions detected in the crude extract did not overlap with those from the extract of the isolate culture of *D. tricolor* [15]. However, we continued the investigation on the fractions, as they showed the bioactivity. Most specialized metabolites showed relatively high intensities in the fractions compared to the crude methanol extract, which suggested that fractions were more concentrated with semi-polar metabolites, mainly due to the removal of the polar primary metabolites (Figure 1A). The LC–MS chromatograms showed that the *n*-hexane fraction exhibited the most distinct chemical compositions, which was observed in the principal coordinate analysis (PCoA) based on the Bray-Curtis similarity between the samples (Figure 1B). For annotation of metabolites, the MS features were organized into a spectral similarity network through the application of the feature-based molecular networking workflow in GNPS [18]. No molecular families were annotated because their spectra did not match the reference spectra; however, the molecular networking analysis revealed that the *n*-hexane fraction contained numerous metabolites that were not abundant in the other fractions (Figure 1C). Thus, we hypothesized that some of these metabolites are the bioactive constituents contributing to the antibacterial activity of the *n*-hexane fraction and performed targeted isolation of the metabolites. In addition, the CH_2_Cl_2_ fraction was applied to the isolation, based on the weak antibacterial activity observed in the bioactivity-guided fractionation.

### 2.3. Isolation and Chemical Characterization of the Compounds

**Figure 2:**
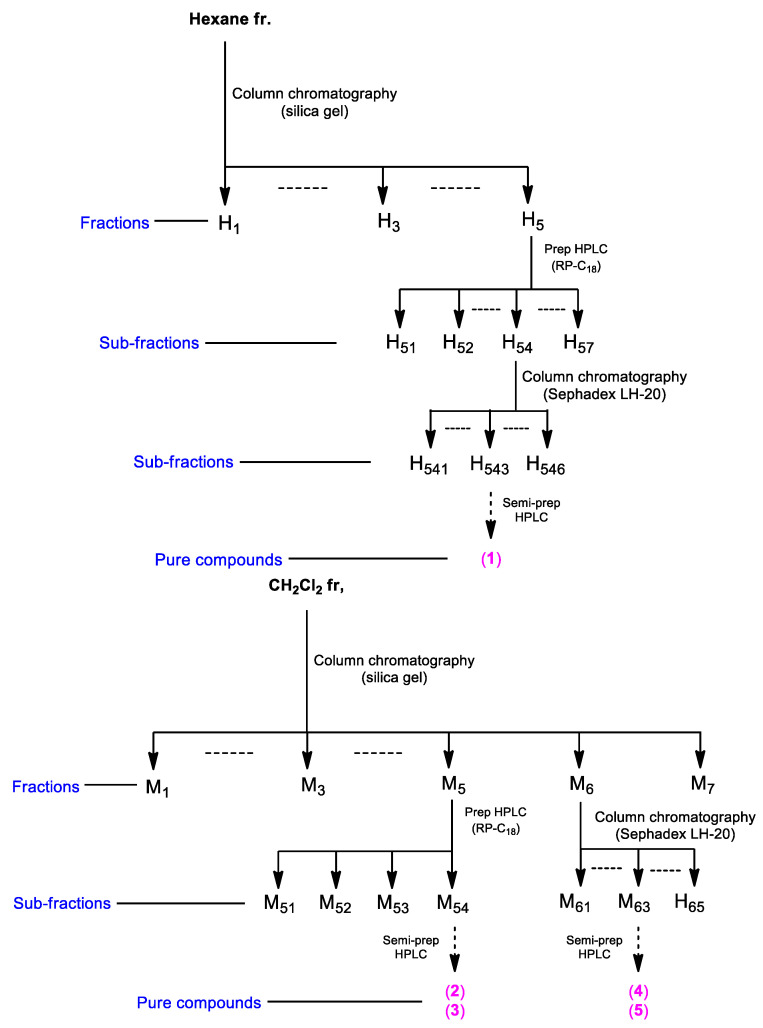
The separation scheme of compounds **1**–**5**.

Based on the LC-MS/MS analysis and bioactivity-guided fractionation, the *n*-hexane- and CH_2_Cl_2_-soluble fractions were subjected to analysis using sequential column chromatography, as well as preparative and semi-preparative HPLC, which resulted in the isolation of five ergosterol derivatives (**1**–**5**) (Figure 2).

**Figure 3:**
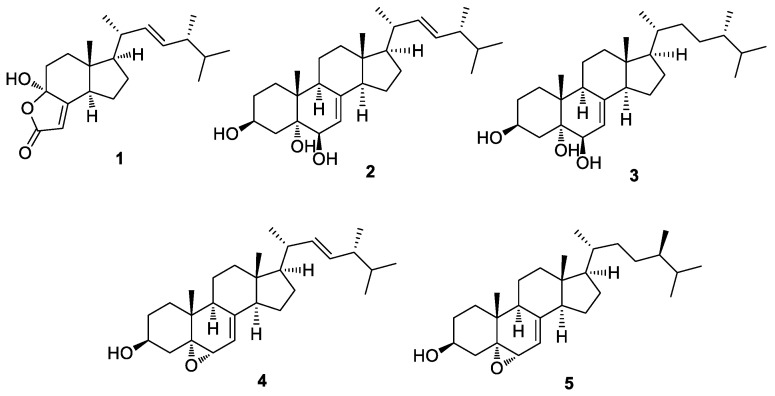
Chemical structures of compounds **1**–**5**.

Compounds **1**–**5** were identified as demethylincisterol A_3_ (**1**) [19], (20*S*,22*E*,24*R*)-ergosta-7,22-dien-3β,5α,6β-triol (**2**) [20], (24*S*)-ergosta-7-ene-3β,5α,6β-triol (**3**) [21], 5α,6α-epoxy-(22*E*,24*R*)-ergosta-7,22-dien-3β-ol (**4**) [22], and 5α,6α-epoxy-(24*R*)-ergosta-7-en-3β-ol (**5**) [23] (Figure 3) by comparing their NMR spectroscopic data with reported values from previously published studies and LC/MS data (Figures S1–S10). This is the first report regarding the presence of the identified ergosterol derivatives (**1**–**5**) in *D. confragosa.*

### 2.4. Evaluation of Antibacterial Activity of the Isolated Compounds against H. pylori

The isolated compounds **1**–**5** were tested for antibacterial activity against *H. pylori* strain 51. Among the isolates from the active fractions, compound **1** showed the most potent anti-*H. pylori* activity with 33.9% inhibition (Table 2); the activity was more potent than that of quercetin, a positive control. Furthermore, compound **3** showed an inhibitory activity comparable to that of quercetin. The other compounds failed to show inhibitory activity. *H. pylori* is a major public health problem worldwide, affecting approximately 50% of the global population [24]. Extermination of *H. pylori* leads to the resolution of both gastritis and gastric ulcers, even gastric cancer [25]; however, clinical failures due to antibiotic resistance are of increasing concern. Thus, there is an urgent need to develop novel antibiotics against *H. pylori* infection. Recent studies have shown that cholesterol, one of the most representative sterols, can play a vital role in virulence and contribute to the intrinsic antibiotic resistance of *H. pylori* [24]. Another sterol cholestenone exhibited antibiotic activity against *H. pylori*, including a clarithromycin-resistant *H. pylori* strain, by suppressing biosynthesis of the cell-wall component cholesteryl-α-ᴅ-glucopyranoside [26]. Based on these findings, we suggest that the antibacterial sterols identified in the present study can potentially serve as clinical antibiotics against *H. pylori* for use; however, further studies on their mechanism and toxicity are needed.

### 2.5. Distribution Analysis of Compound ***1*** in D. confragosa and Other Fungi

**Figure 4:**
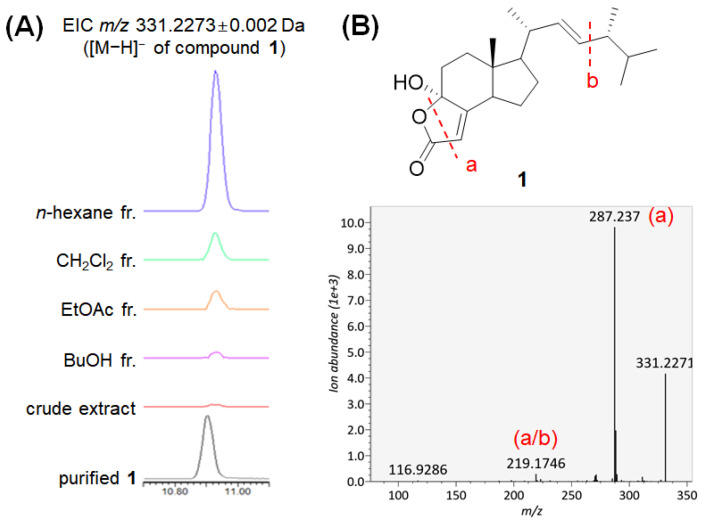
(**A**) Detection of compound **1** in the crude extract and fractions of *D. confragosa*. EICs of the extract and fractions are in the same scale; the peak height represents the relative abundance of **1**. (**B**) The MS/MS spectrum of compound **1**. The putative substructural annotations on the fragment ions are shown.

The relative abundance of bioactive compound **1** in each fraction was analyzed based on the LC–MS data of the fractions. The extracted ion chromatogram of m/z 331.2273 representing the deprotonated ion of **1** revealed that compound **1** was significantly abundant in the *n*-hexane fraction (Figure 4A), which might contribute to the high bioactivity of the fraction. We further searched the MS/MS spectrum of compound **1** (Figure 4B) against all publicly available MS/MS datasets deposited in the GNPS MassIVE database through MASST [27] to determine the distribution of this antibacterial sterol among the fungal species. In the MASST search, 20 datasets containing MS/MS spectra matched the spectrum of **1**. After removing the matches showing Δ*m/z* > 0.02 Da, four datasets remained, all of which were from fungi. Among these four datasets, three were available for metadata and two were obtained from our previous studies. Compound **1** was present in the fungal cell extracts of *Fusarium graminearum* (dataset MSV000084977) [28] and fungal culture extracts from eight species in our previous Polyporaceae dataset (MSV000085974) [15], *Cerrena unicolor*, *Coriolopsis strumosa*, *Daedaleopsis tricolor*, *Polyporus brumalis*, *Microporus vernicipes*, *Trametes orientalis*, *Trametes suaveolens*, and *Trametopsis cervina*. Interestingly, the extract of *D. conragosa* in the Polyporaceae dataset did not contain **1**, which suggests that the biosynthesis of **1** was not activated under the laboratory culture conditions used in this study. Another dataset, MSV000085071, which contains data from mushroom samples collected during the Mount Pisgah mushroom festival in September 2019, contained 66 files matched to the spectrum of **1**. The deposited metadata provided 48 species names for the matched files (Table 3). This result revealed the wide contribution of compound **1** in the kingdom Fungi.

## 3. Materials and Methods

### 3.1. General Experimental Procedure

Optical rotations were measured using a Jasco P-1020 polarimeter (Jasco, Easton, MD, USA). Nuclear magnetic resonance (NMR) spectra were recorded using a Bruker Avance III HD 850 NMR spectrometer operating at 850 MHz (^1^H), with chemical shifts given in ppm (δ). Preparative high-performance liquid chromatography (HPLC) was performed using a Waters 1525 Binary HPLC pump with a Waters 996 Photodiode Array Detector (Waters Corporation, Milford, CT, USA) and an Agilent Eclipse C18 column (250 mm × 21.2 mm, 5 μm; flow rate: 5 mL/min; Agilent Technologies). Semi-preparative HPLC was performed using a Shimadzu Prominence HPLC System with SPD-20A/20AV Series Prominence HPLC UV-Vis Detectors (Shimadzu, Tokyo, Japan). LC/MS analysis was performed on an Agilent 1200 Series HPLC system (Agilent Technologies, Santa Clara, CA, USA) equipped with a diode array detector and a 6130 Series ESI mass spectrometer using an analytical Kinetex C18 Å column (100 mm × 2.1 mm, 5 μm; flow rate: 0.3 mL/min; Phenomenex). Spots were detected on a TLC plate under UV light or following heating after spraying with anisaldehyde–sulfuric acid. Silica gel 60 (230–400 mesh; Merck, Darmstadt, Germany) was used for column chromatography. Sephadex LH-20 (Pharmacia, Uppsala, Sweden) was used as the packing material for molecular sieve column chromatography. Merck precoated silica gel F254 plates and RP-18 F254s plates were used for thin-layer chromatography (TLC).

### 3.2. Mushroom Material

Fresh fruiting bodies of *D. confragosa* were collected at the Herb Garden of the College of Pharmacy, Hanyang University, Ansan, Korea in August 2019. DNA sequences were analyzed for identifying the species of this material using a modified method [29]. PCR amplification was performed using the fungal-specific PCR primers ITS1 and ITS4 for the internal transcribed spacer (ITS) region using the modified method [30]. The homology of this region sequence was matched with *Deadaleopsis confragosa* with the highest matching scores in the NCBI BLAST database. A voucher specimen (SKKU-DJBS-2019-08) of the mushroom was deposited at the herbarium of the School of Pharmacy, Sungkyunkwan University, Korea.

### 3.3. Extraction of D. Confragosa and Solvent Partition

Completely dried *D. confragosa* fruiting bodies (2 kg) were extracted with 80% methanol under reflux three times (6.0 L × 3) and subsequently filtered. The filtrate was subsequently evaporated in vacuo to obtain a crude methanol extract (59.3 g). The resultant extract was dissolved in distilled water (700 mL) and then solvent partitioned with hexane, CH_2_Cl_2_, EtOAc, and *n*-BuOH (700 mL × 3). Four layers with increasing polarity were obtained: hexane (3.4 g), CH_2_Cl_2_ (6.5 g), EtOAc (0.7 g), and *n*-BuOH-soluble (2.6 g) fractions.

### 3.4. LC–MS/MS Analysis

The crude extract and fractions were dissolved in 50% methanol at a concentration of 1.0 mg/mL and filtered through a 0.22 μm PTFE syringe filter (Altoss, Sejong, Korea). LC–MS/MS analysis was performed using a Waters Acquity I-Class UPLC system (Waters Co., Milford, MA, USA) hyphenated to a Waters VION IMS QTOF mass spectrometer (Waters MS Technologies, Manchester, UK). Chromatographic separation was performed on a Waters Acquity UPLC BEH C_18_ (100 mm × 2.1 mm, 1.7 μm) column maintained at 40 °C. Elution was performed with a mixture of 0.1% formic acid in H_2_O (A) and MeCN (B) in a linear gradient of 10–100% B (0–12 min) followed by 3 min of washing with 100% B and 3 min of column re-conditioning with 10% B at a flow rate of 0.3 mL/min. The samples (2.0 μL of injection volume) were analyzed in the negative ion mode. MS/MS spectra were acquired in the MS^E^ data-independent acquisition (DIA) mode with a low collision energy of 6 eV for the detection of precursor ions and high collision energy of 20–40 eV for fragmentation. The [M−H]^−^ ion of leucine enkephalin at *m*/*z* 554.2615 was used as the lock mass to ensure mass accuracy and reproducibility. Chromatographic deconvolution and feature finding were performed using MS-DIAL 4.60 [31], and the resulting feature table and MS/MS spectral list were subjected to the feature-based molecular networking workflow [18] of the Global Natural Products Social Molecular Networking (GNPS; https://gnps.ucsd.edu, accessed on 5 December 2021) environment [32].

Raw LC-MS/MS data and the result files from MS-DIAL processing were deposited in the MassIVE (https://massive.ucsd.edu, accessed on 5 December 2021) with the accession no. MSV000089017.

The MS/MS spectral network can be accessed via the following link: https://gnps.ucsd.edu/ProteoSAFe/status.jsp?task=66d2218f575849a8aecbdb379ae99386, accessed on 5 December 2021.

The experimental spectrum of **1** was searched against public datasets deposited in GNPS MassIVE via MASST [27]. The MASST search results can be accessed via https://gnps.ucsd.edu/ProteoSAFe/status.jsp?task=f0e9eb1240894f838b5079f4f34bc5c6, accessed on 5 December 2021.

### 3.5. Isolation of Compounds from the Hexane and CH_2_Cl_2_ Fractions

The hexane-soluble fraction (3.4 g) was loaded onto a silica gel chromatography column and fractionated using a gradient solvent system of hexane-EtOAc (30:1–1:1, *v*/*v*) to yield five fractions (H1–H5). Seven subfractions (H51–H57) were obtained from the fraction H5 (486 mg) by preparative reversed-phase HPLC using acetonitrile–H_2_O (2:1–1:0, *v*/*v*, gradient solvent system, flow rate: 5 mL/min). The H54 subfraction (109 mg) was applied to a Sephadex LH-20 column using CH_2_Cl_2_–MeOH (1:1, *v*/*v*) to obtain six subfractions (H541-H546). Compound **1** (1.0 mg) was isolated from subfraction H543 (12 mg) via semi-preparative reversed-phase HPLC with 88% MeOH/H_2_O (isocratic solvent system, flow rate: 2 mL/min). The CH_2_Cl_2_ fraction (6.5 g) was loaded onto a silica gel chromatography column and fractionated using a gradient solvent system of CH_2_Cl_2_–MeOH (29:1–1:1, *v*/*v*) to yield seven fractions (M1–M7). Four subfractions (M51–M54) were obtained from fraction M5 (143 mg) by preparative reversed-phase HPLC using acetonitrile-H_2_O (70–100% MeOH/H_2_O, *v*/*v*, gradient solvent system, flow rate: 5 mL/min). Compounds **2** (0.8 mg, *t*_R_ = 40.0 min) and **3** (0.5 mg, *t*_R_ = 44.0 min) were isolated from subfraction M54 (34 mg) via semi-preparative reversed-phase HPLC using 85% MeOH/H_2_O (isocratic solvent system, flow rate: 2 mL/min). Five subfractions (M61–M65) were obtained from fraction M6 (140 mg) using a Sephadex LH-20 column using CH_2_Cl_2_–MeOH (1:2, *v*/*v*). Finally, subfraction M63 (14 mg) was purified by semi-preparative reversed-phase HPLC with 55% CH_3_CN/H_2_O (isocratic solvent system, flow rate: 2 mL/min) to afford compounds **4** (1.0 mg, *t*_R_ = 33.0 min) and **5** (0.7 mg, *t*_R_ = 38.0 min).

### 3.6. Helicobacter Pylori Culture

A clinical strain of *H. pylori* 51 was provided by the *H. pylori* Korean Type Culture Collection, School of Medicine, Gyeongsang National University, Korea. The strain was grown and maintained on Brucella agar medium (BD Co., Sparks, MD, USA) supplemented with 10% horse serum (Gibco, New York, USA). The culture conditions were 37 °C, 100% humidity, and 10% CO_2_ for 2–3 days.

### 3.7. Anti-Helicobacter Pylori Activity

Twenty microliters of the bacterial colony suspension equivalent to 2 × 10^8^–3 × 10^8^ cfu/mL and 20 µL of test samples or controls were added to Brucella broth medium supplemented with 10% horse serum to each well in a 6-well plate. The final volume was 2 mL, and the final concentrations were 100 μg/mL and 100 μM for the fractions and compounds, respectively. After 24 h of incubation at 37 °C, growth was assessed by measuring the optical density at 600 nm using a spectrophotometer. Quercetin and metronidazole were purchased from Sigma (St. Louis, MO, USA) and used as positive controls.

## 4. Conclusions

In this study, we isolated and characterized the potential anti-*H. pylori* compounds, five ergosterol derivatives (**1**–**5**) from the methanolic extracts of *D. confragosa* fruiting bodies via bioactivity-guided fractionation and LC–MS/MS profiling. Their structures were established using NMR spectroscopy and LC-MS analysis. In the anti-*H. pylori* activity test, we demonstrated that compound **1** showed the most potent anti-*H. pylori* activity with 33.9% inhibition and compound **3** showed inhibitory activity comparable to that of quercetin, a positive control. Distribution analysis of compound **1** by LC–MS/MS-based analysis indicated the wide distribution of compound **1** in the kingdom Fungi. Based on these findings, we conclude that active demethylincisterol A_3_ (**1**) may be used to develop novel antibiotics against *H. pylori*.

## Figures and Tables

**Table 1:** Anti-*H. pyroli* activity of the MeOH extract and fractions derived from the solvent partitioning.

Sample	Concentrations	Inhibition (%)
Methanolic extract	100 μg/mL	22.4
*n*-hexane fraction		38.2
CH_2_Cl_2_ fraction		23.3
EtOAc fraction		16.0
BuOH fraction		15.6
Quercetin ^a^	100 μM	22.2
Metronidazole ^a^		73.5

^a^ Positive controls.

**Table 2:** Anti-*H. pyroli* activity of compounds **1**–**5**.

Compound	Concentrations	Inhibition (%)
**1**	100 μM	33.9
**2**		8.6
**3**		18.5
**4**		6.8
**5**		3.4
Quercetin ^a^	100 μM	22.2
Metronidazole ^a^		73.5

^a^ Positive controls.

**Table 3:** Fungal species found to contain compound **1**, according to the MASST analysis.

Dataset	Species	Family
MSV000084977	*Fusarium graminearum*	Nectraiaceae
MSV000085071	*Leucoagaricus atrodisca*	Agaricaceae
	*Lepiota spheniscuspora*	
	*Amanita pantherinoides*	Amanitaceae
	*Amanita protecta*	
	*Phylloporus arenicola*	Boletaceae
	*Suillelus amygdalinus*	
	*Bondarzewia occidentalis*	Bondarzewiaceae
	*Cantharellus californicus*	Cantharellaceae
	*Cantharellus subalbidus*	
	*Craterellus tubaeformis*	
	*Clavariadelphus mucronatus*	Clavariadelphaceae
	*Clavariadelphus occidentalis*	
	*Clavulina cinerea*	Clavulinaceae
	*Cortinarius alboglobosus*	Cortinariaceae
	*Cortinarius albogfragrans*	
	*Cortinarius alboviolaceus*	
	*Cortinarius anomalus*	
	*Cortinarius cinnamomeus*	
	*Cortinarius collinitus*	
	*Cortinarius caperatus*	
	*Cortinarius causticus*	
	*Cortinarius croceus*	
	*Clitopilus hobsonii*	Entolomataceae
	*Gomphus vlavatus*	Gomphaceae
	*Ramaria araispora* var. *araiospora*	
	*Ramaria formosa*	
	*Ramaria gelatonosa* var. *oregonensis*	
	*Ramaria rubripermanens*	
	*Ramaria sandaracina* var. *sandaracina*	
	*Hydnum umbilicatum*	Hydnaceae
	*Armillaria solidipes*	Physalacriaceae
	*Polyporus leptocephalus*	Polyporaceae
	*Trametes hirsuta*	
	*Trametes versicolor*	
	*Coprinopsis atramentaria*	Psathyrellaceae
	*Lactarius aestivus*	Russulaceae
	*Lactarius rubrilacteus*	
	*Lactarius scrobiculatus*	
	*Russula americana*	
	*Russula xerampelina*	
	*Pholiota flammans*	Strophariaceae
	*Clitocybe nuda*	Tricholomataceae
	*Collybia cirrhata*	
	*Leucopaxillus albissimus*	
	*Tricholoma inamoenum*	
	*Tricholoma mutabile*	
	*Tricholoma pardinum*	
	*Tricholoma portentosum*	
MSV000085974	*Cerrena unicolor*	Polyporaceae
	*Coriolopsis strumose*	
	*Daedaleopsis tricolor*	
	*Microporus vernicipes*	
	*Polyporus brumalis*	
	*Trametes orientalis*	
	*Trametes suaveolens*	
	*Trametopsis cervina*	

## Supplementary Materials

The following supporting information can be downloaded at: https://www.mdpi.com/article/10.3390/molecules27061865/s1, Figure S1: ^1^H-NMR spectrum of compound **1** (in CD_3_OD); Figure S2: LC/MS data of compound **1**; Figure S3: ^1^H-NMR spectrum of compound **2** (in CD_3_OD); Figure S4: LC/MS data of compound **2**; Figure S5: ^1^H-NMR spectrum of compound **3** (in CD_3_OD); Figure S6: LC/MS data of compound **3**; Figure S7: ^1^H-NMR spectrum of compound **4** (in CDCl_3_); Figure S8: LC/MS data of compound **4**; Figure S9: ^1^H-NMR spectrum of compound **5** (in CDCl_3_); Figure S10: LC/MS data of compound **5**.
