# Supplementary material for: Identification of Antibacterial Sterols from Korean Wild Mushroom Daedaleopsis confragosa via Bioactivity- and LC-MS/MS Profile-Guided Fractionation
Source: Molecules. 2022 Mar 14;27(6):1865. doi: 10.3390/molecules27061865 (PMC8954928; doi:10.3390/molecules27061865)
Supplement: Supplementary file 1 [file molecules-27-01865-s001.zip › molecules-1582673-supplementary.pdf]

Supplementary Materials

Identification of Antibacterial Sterols from Korean Wild Mushroom *Daedaleopsis confragosa* via Bioactivity- and LC-MS/MS profile-guided Fractionation

Myung Woo Na [1], Eunjin Lee [2], Dong-Min Kang [3], Se Yun Jeong [1], Rhim Ryoo [4], Chul-Young Kim [5], Mi-Jeong Ahn [3], Kyo Bin Kang,[2,*] and Ki Hyun Kim[1,*]

1 School of Pharmacy, Sungkyunkwan University, Suwon 16419, Korea; myeong500@naver.com (M.W.N.); dlawktkark@naver.com (S.Y.J.)

2 Research Institute of Pharmaceutical Sciences, College of Pharmacy, Sookmyung Women's University, Seoul 04310, Korea; dmswls8180@sookmyung.ac.kr (E.L.)

3 College of Pharmacy and Research Institute of Pharmaceutical Sciences, Gyeongsang National University, Jinju 52828, Korea; kdm7105@gnu.ac.kr (D.M.K.); amj5812@gnu.ac.kr (M.J.A.)

4 Special Forest Products Division, Forest Bioresources Department, National Institute of Forest Science, Suwon 16631, Korea; rryoo@korea.kr (R.R.)

5 College of Pharmacy, Hanyang University, Ansan 15588, Korea; chulykim@hanyang.ac.kr (C.Y.K.)

* Correspondence: khkim83@skku.edu (K.H.K.); Tel.: +82-31-290-7700 (K.H.K.); kbkang@sookmyung.ac.kr (K.B.K.); Tel: +82-2-2077-7103 (K.B.K.)

Figure S1 : ^{1}H-NMR spectrum of compound **1** (in CD_3OD)
Figure S2 : LC/MS data of compound **1**
Figure S3 : ^{1}H-NMR spectrum of compound **2** (in CD_3OD)
Figure S4 : LC/MS data of compound **2**
Figure S5 : ^{1}H-NMR spectrum of compound **3** (in CD_3OD)
Figure S6 : LC/MS data of compound **3**
Figure S7 : ^{1}H-NMR spectrum of compound **4** (in $CDCl_3$)
Figure S8 : LC/MS data of compound **4**
Figure S9 : ^{1}H-NMR spectrum of compound **5** (in $CDCl_3$)
Figure S10 : LC/MS data of compound **5**

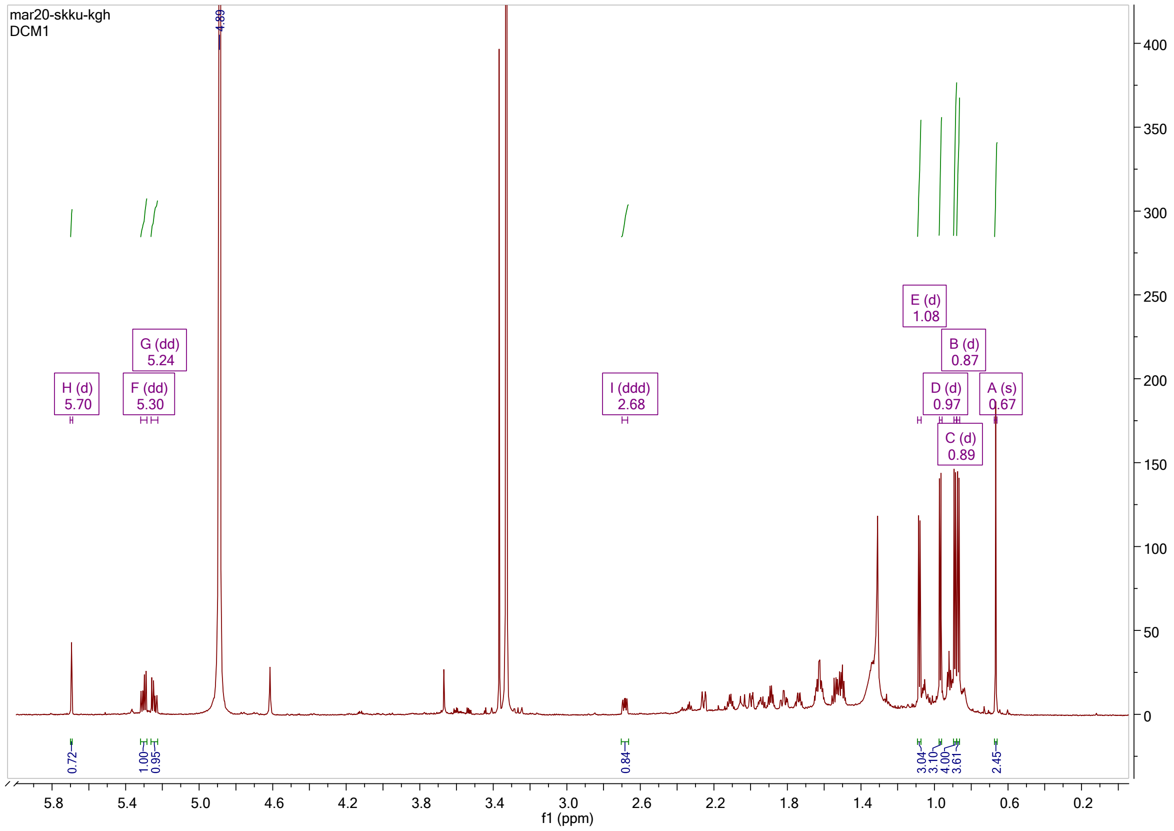

Figure S1 : ^{1}H-NMR spectrum of compound **1** (in CD_3OD)

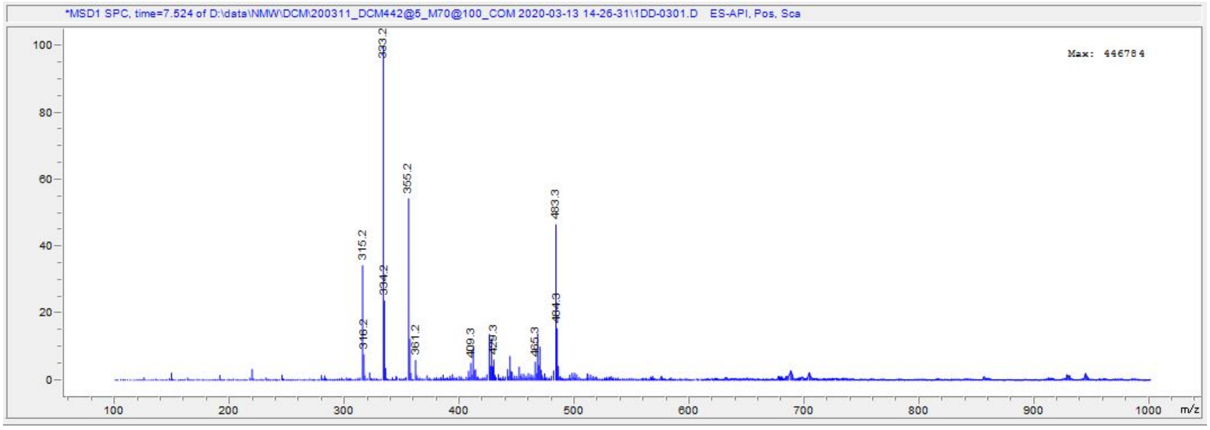

Figure S2 : LC/MS data of compound **1**

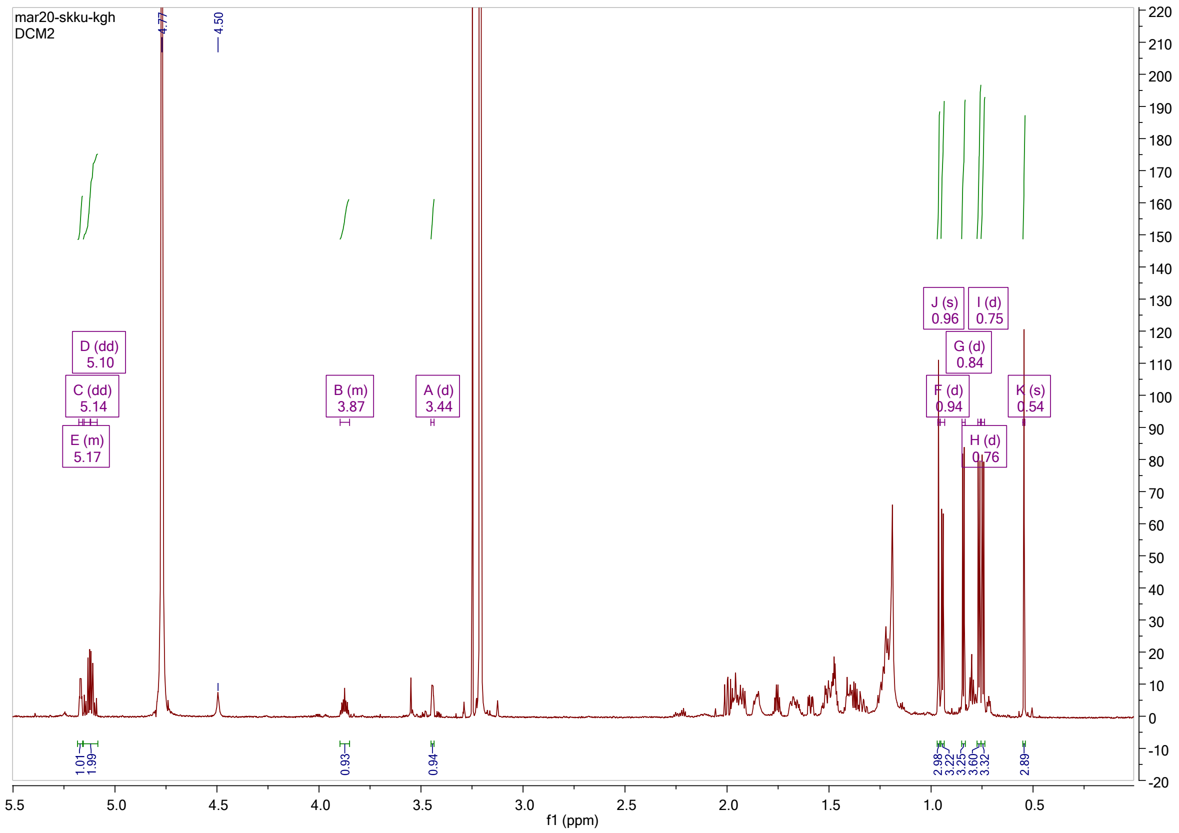

Figure S3 : ^{1}H-NMR spectrum of compound **2** (in CD_3OD)

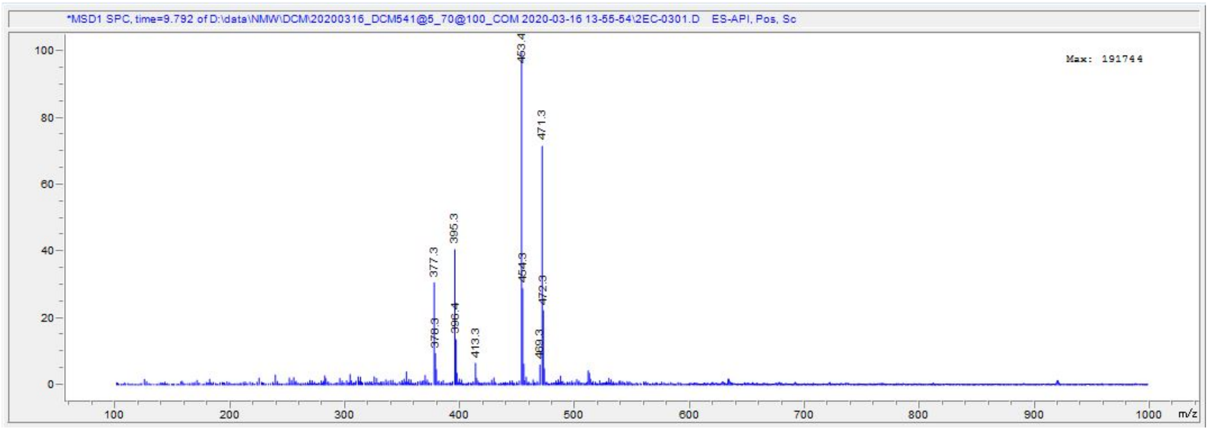

Figure S4 : LC/MS data of compound **2**

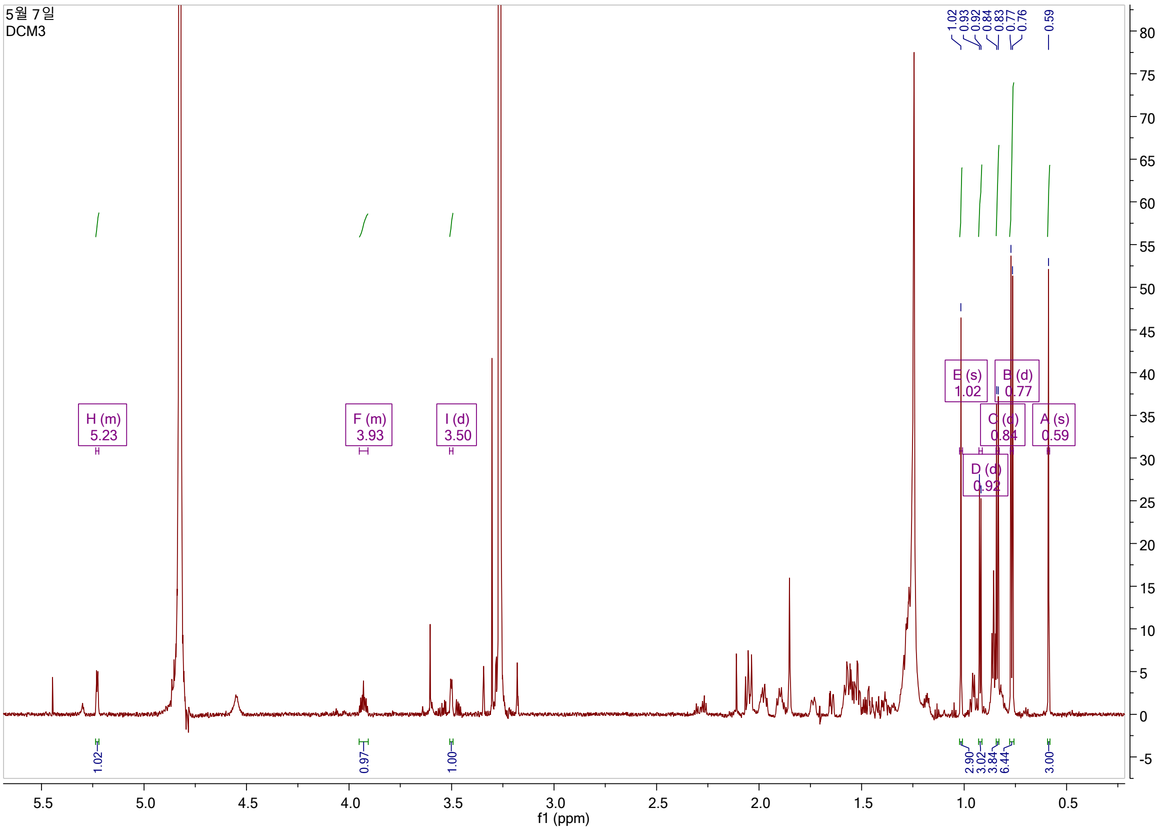

Figure S5 : ^{1}H-NMR spectrum of compound **3** (in CD_3OD)

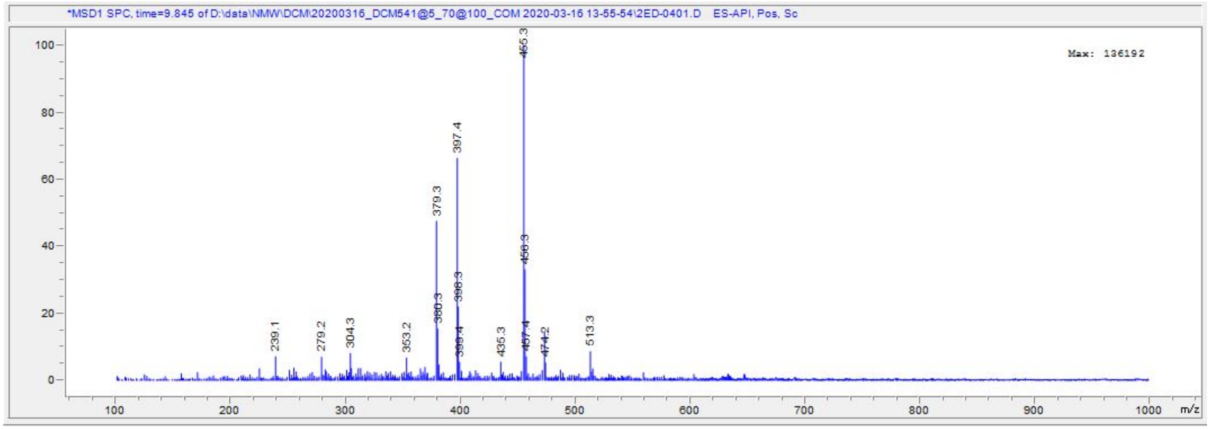

Figure S6 : LC/MS data of compound **3**

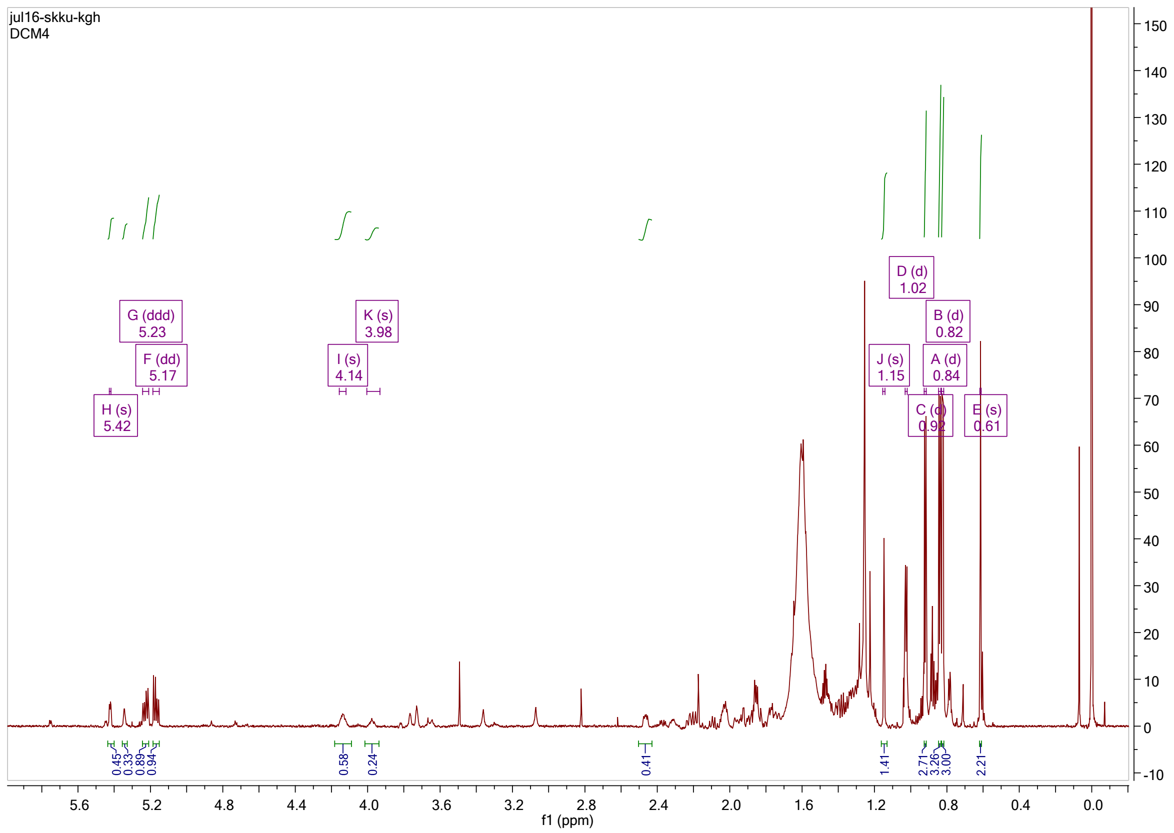

Figure S7 : ^{1}H-NMR spectrum of compound **4** (in $CDCl_3$)

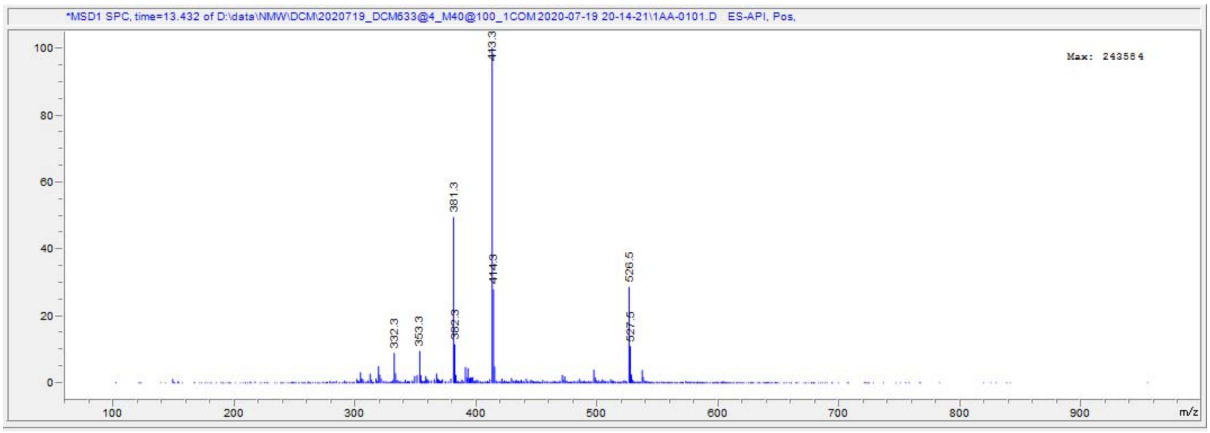

Figure S8 : LC/MS data of compound **4**

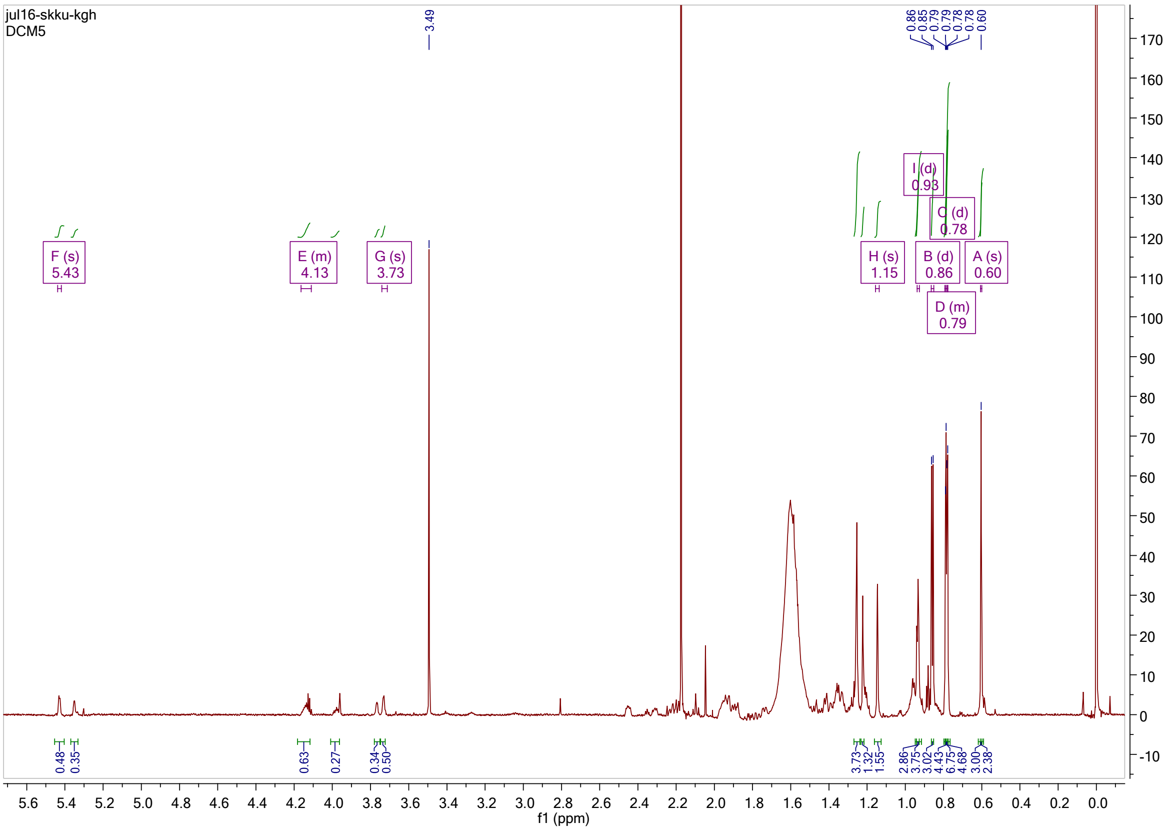

Figure S9 : ^{1}H-NMR spectrum of compound **5** (in $CDCl_3$)

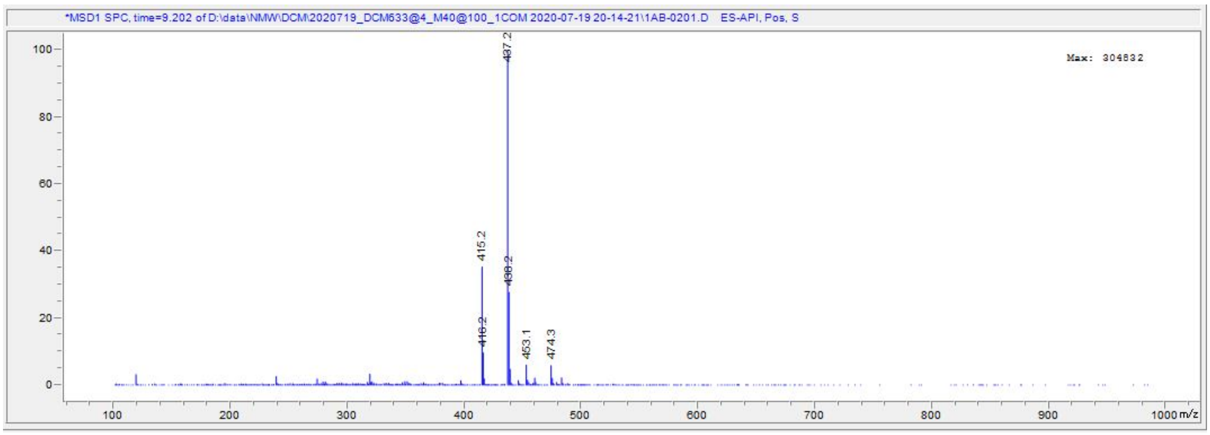

Figure S10 : LC/MS data of compound **5**
